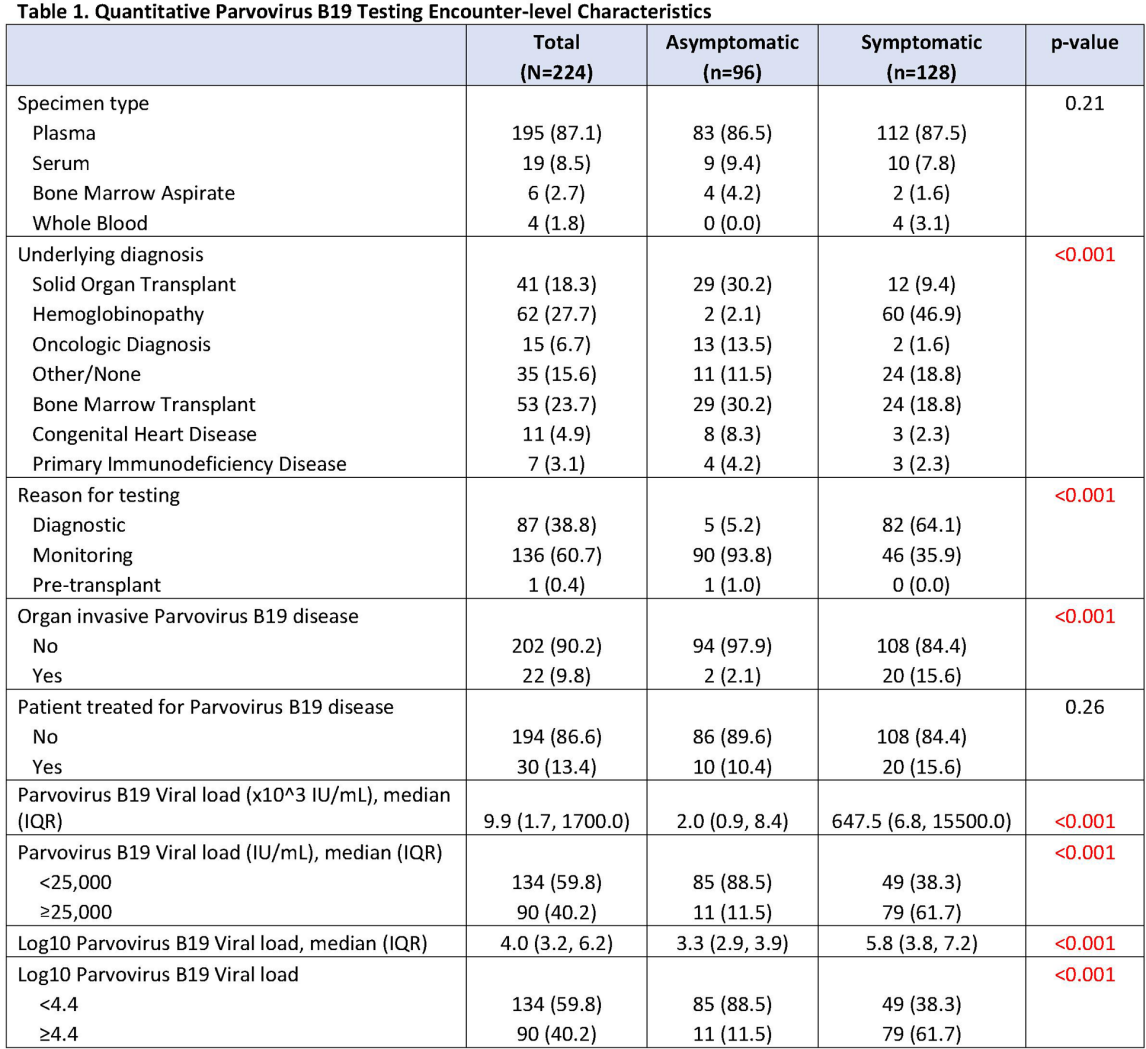# 314 Characteristics of outpatient parenteral antimicrobial therapy (OPAT) delivery within Veterans Affairs Medical Centers

**DOI:** 10.1017/ash.2026.10665

**Published:** 2026-06-23

**Authors:** Elizabeth Tocco, Erin Nicholson, Josalyn Curl, Vincent Ferreira, Duc Nguyen, Ankhi Dutta

**Affiliations:** 1 Texas Children’s Hospital; 2 Baylor College of Medicine

## Abstract

**Background:** Parvovirus B19 causes a spectrum of illness in children, from asymptomatic infection to severe organ-invasive disease, particularly in immunocompromised hosts. Current infection prevention guidelines recommend prolonged isolation for patients with chronic infection, yet no validated quantitative PCR threshold exists to guide discontinuation of precautions. This uncertainty may lead to extended isolation and resource utilization. Objective: To evaluate the association between quantitative parvovirus B19 PCR levels and clinical manifestations in pediatric patients and explore implications for infection control practices. **Methods:** We conducted a retrospective observational study of patients aged 0–18 years who underwent quantitative parvovirus B19 PCR testing in a quaternary care hospital system from January–December 2024. Demographics, clinical features, and treatment were abstracted from the electronic medical record. Encounter-level and patient-level characteristics were summarized using Fisher’s exact and Kruskal-Wallis tests. Multivariable generalized estimating equation (GEE) models assessed factors associated with symptomatic infection. Optimal viral load cut-off associated with symptomatology was determined using Youden’s index. A patient with symptomatic infection was defined as having one or more signs/symptoms associated with clinical parvovirus B19 infection (fever, rash, arthropathy, cytopenias, carditis, hepatitis, etc.) that was not explained by a pre-existing underlying condition. **Results:** Among 103 patients (median age 7 years [IQR 4–11]), 224 encounters were analyzed; 57% were symptomatic (Table 1). Median viral load was 647,500 IU/mL (IQR 6,800–15,500,000) in symptomatic encounters versus 2,000 IU/mL (IQR 900–8,400) in asymptomatic (p<0.001). An optimal threshold of ?25,000 IU/mL (log10 ?4.4) was strongly associated with symptoms (adjusted OR [aOR] 6.54, 95% CI 1.98–21.64; p=0.002). Additional predictors associated with symptoms included hemoglobinopathy (aOR <700; p<0.001) and diagnostic testing indication (aOR 155.01, p<0.001). Viral load was not associated with organ-invasive disease or treatment response. **Conclusions:** Higher parvovirus B19 viral loads are associated with symptomatic infection, but not with organ invasion or treatment initiation. A threshold of ?25,000 IU/mL may inform future infection prevention guidance, potentially helping to modify isolation practices and improving resource utilization.

**Figure:**